# A study on the diversity of phlebotomine sand flies (Diptera, Psychodidae) in karstic limestone areas in Vientiane Province, Laos, with a description of two new species of *Sergentomyia* França and & Parrot

**DOI:** 10.1186/s13071-024-06444-w

**Published:** 2024-09-11

**Authors:** Khamsing Vongphayloth, Fano José Randrianambinintsoa, Khaithong Lakeomany, Nothasine Phommavanh, Tavun Pongsanarm, Veaky Vungkyly, Phonesavanh Luangamath, Somsanith Chonephetsarath, Paul T. Brey, Jérôme Depaquit

**Affiliations:** 1 Laboratory of Vector-Borne Diseases, Institut Pasteur du Laos, Samsenthai Road, Ban Kao-Gnot, Sisattanak District, 3560 Vientiane, Lao PDR; 2 Université de Reims Champagne-Ardenne, Univ Rouen Normandie, Normandie Univ, ESCAPE, Reims, France; 3 USC ANSES Petard, Reims, France; 4 Laboratoire de Parasitologie-Mycologie, Pôle de Biologie Territoriale, CHU, Reims, France

**Keywords:** Ecology, *Phlebotomus*, *Sergentomyia*, *Idiophlebotomus*, *Chinius*, *Grassomyia*, Lao PDR

## Abstract

**Background:**

Southeast Asia is well known as a hotspot of biodiversity. However, very little is known about cave-dwelling hematophagous insects that are medically important. Taxonomic knowledge and ecology of phlebotomine sand flies are very poorly studied in Laos, as well as in other countries in the region. Herein, we report species diversity data and some notes on the ecology of the detected species from these karstic limestone areas of Laos.

**Methods:**

Phlebotomine sand flies were collected using Centers for Disease Control and Prevention (CDC) light traps from limestone cave locations in three districts of Vientiane Province, Laos. Both morphological and molecular techniques were used for sand fly identification. Species diversity and abundance were analyzed according to sites, locations, collection seasons, and trapping positions.

**Results:**

A total of 6564 sand flies, of which 5038 were females and 1526 were males, were morphologically identified into 20 species belonging to five genera (*Chinius*, *Idiophlebotomus*, *Phlebotomus*, *Sergentomyia*, and *Grassomyia*). The most abundant species were *Chinius eunicegalatiae*, *Phlebotomus stantoni*, *Sergentomyia hivernus*, *Se. siamensis*, and *Idiophlebotomus longiforceps*. Cytochrome b analysis results supported the morphological identification and revealed that *Se. siamensis* was separated from other members of the *Se. barraudi* group. Two new species, *Se. dvoraki* n. sp. and *Se. marolii* n. sp., were described. Sand fly density was generally high except in a cave in Vangvieng, with species richness ranging from 14 to 18 across different caves. Outside caves had higher species richness (R = 20) and diversity (*H* = 2.50) than cave entrances (R = 18, *H* = 2.41) and interiors (R = 16, *H* = 2.13). Seasonal variations showed high sand fly density in Feung and Hinheup during both dry and rainy seasons, while Vangvieng had a notable decrease in density during the dry season (D = 6.29).

**Conclusions:**

This study revealed that the diversity of phlebotomine sand fly fauna in Laos, particularly in karstic limestone areas, is greater than previously known. However, the taxonomic status of many species in Laos, as well as Southeast Asia, still needs more in-depth study using both morphological characters and molecular methods. Many species could be found from inside, at the entrance, and outside of caves, indicating a wide range of host-seeking behavior or possible natural breeding in the karstic cave areas.

**Graphical abstract:**

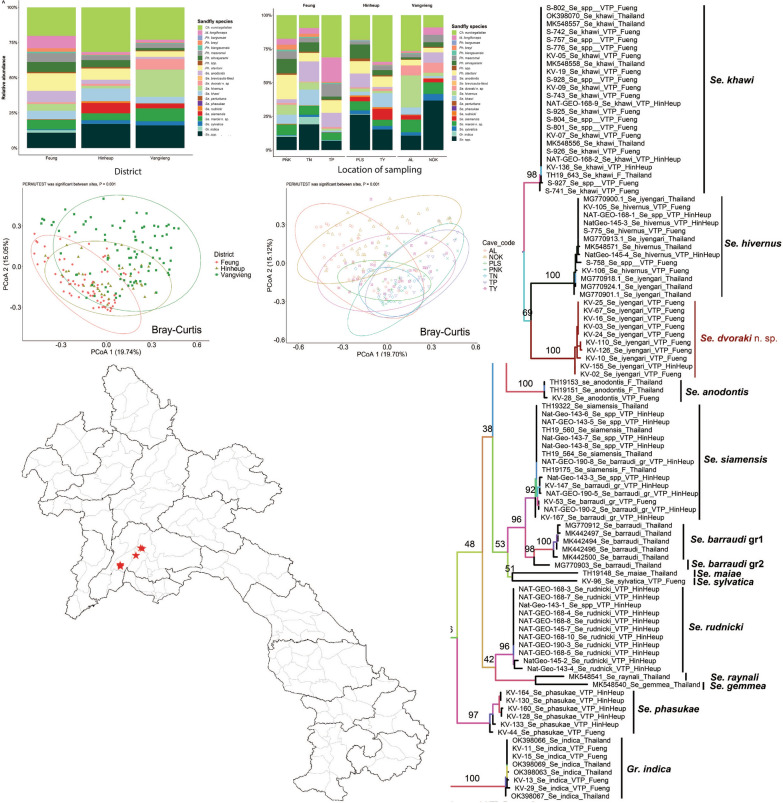

**Supplementary Information:**

The online version contains supplementary material available at 10.1186/s13071-024-06444-w.

## Background

Vector-borne diseases (VBDs) are the second most common cause of emerging infectious disease (EID) events after zoonotic pathogens [1]. Over the past few decades, there has been significant emergence and re-emergence of several VBDs, including malaria, leishmaniasis, dengue, yellow fever, Zika, chikungunya, and plague [2–4]. The emergence of new and resurgent known vector-borne pathogens can be associated with several factors, including adaptation to and changes in microorganisms, habitat, globalization, tourism, and travel [5, 6].

Karstic areas and cave ecosystems are specific environmental conditions that provide suitable places for insects and harbor many different opportunistic pathogens, such as viruses, bacteria, and fungi [7, 8]. Many of these pathogens infect cave-dwelling vertebrates, especially bats [9–13]. Some of these viruses can be transmitted from one vertebrate host to another via hematophagous arthropod vectors (e.g., mosquitoes, sand flies, bat flies, and biting midges) [14–16]. Humans and other animals that visit these environments are at risk of exposure to arthropod vectors in caves and in surrounding areas. Additionally, visitors themselves may inadvertently introduce pathogens to these areas where vectors are present. This introduces the possibility of pathogens being introduced to cave ecosystems through human activities, highlighting the need for careful consideration and monitoring of potential disease transmission pathways in these environments.

In Southeast Asia (SE Asia), cave visits are increasingly popular for many reasons, whether by local populations looking for resources, for economic purposes such as ecotourism, or for spiritual purposes (cave-dwelling monks). Therefore, such growing human incursions into caves may increase the risk of exposure and spillover of emerging pathogens that circulate among cave-dwelling vertebrates and vectors. SE Asia is a well-known biodiversity hotspot [17]. However, very little is known about cave-dwelling hematophagous insects that are medically important, such as mosquitoes (Culicidae), sand flies (Psychodidae), and biting midges (Ceratopogonidae). Taxonomic knowledge of sand flies is very poor among countries in SE Asia. The last revision of sand flies in this region was made by Lewis in 1978 [18]. The first autochthonous human case of leishmaniasis was reported in Thailand in 1996, followed by several cases caused by *Leishmania* (*Mundinia*) *martiniquensis* (formerly identified as *Leishmania siamensis*) [19–21]. According to these results, Thailand is the only country in the region with advanced studies on sand flies. *Leishmania* DNA has been detected in the *Sergentomyia barraudi* and *Sergentomyia iyengari* sensu Raynal 1936 groups, notably including *Sergentomyia khawi*, which was probably misidentified as *Sergentomyia gemmea* [22]. However, polymerase chain reaction (PCR) alone cannot conclusively prove whether an insect can transmit pathogens. Additional methods are necessary to confirm the insect’s role as a disease vector, as PCR detects pathogen genetic material but does not demonstrate transmission capability.

In Laos, before this study, only one cavernicolous species of sand fly belonging to the genus *Chinius* Leng had been reported, in 2010 [23], while two other species of *Phlebotomus* and five species of *Sergentomyia* were reported in 1962 by Quate [24]. In 2019, one more species of *Sergentomyia* from Laos was reported [22]. To our knowledge, there are no data on the diversity of cavernicolous or karstic insects of medical or veterinary interest in Laos. Thus, to fill this gap, we investigated sand flies in karstic areas of Vientiane Province in Laos in 2019, and two more new species were described [25]. Here, we report additional data on species diversity and some notes on the ecology of the detected species in karstic limestone areas of Laos.

## Methods

### Study sites

**Fig. 1:**
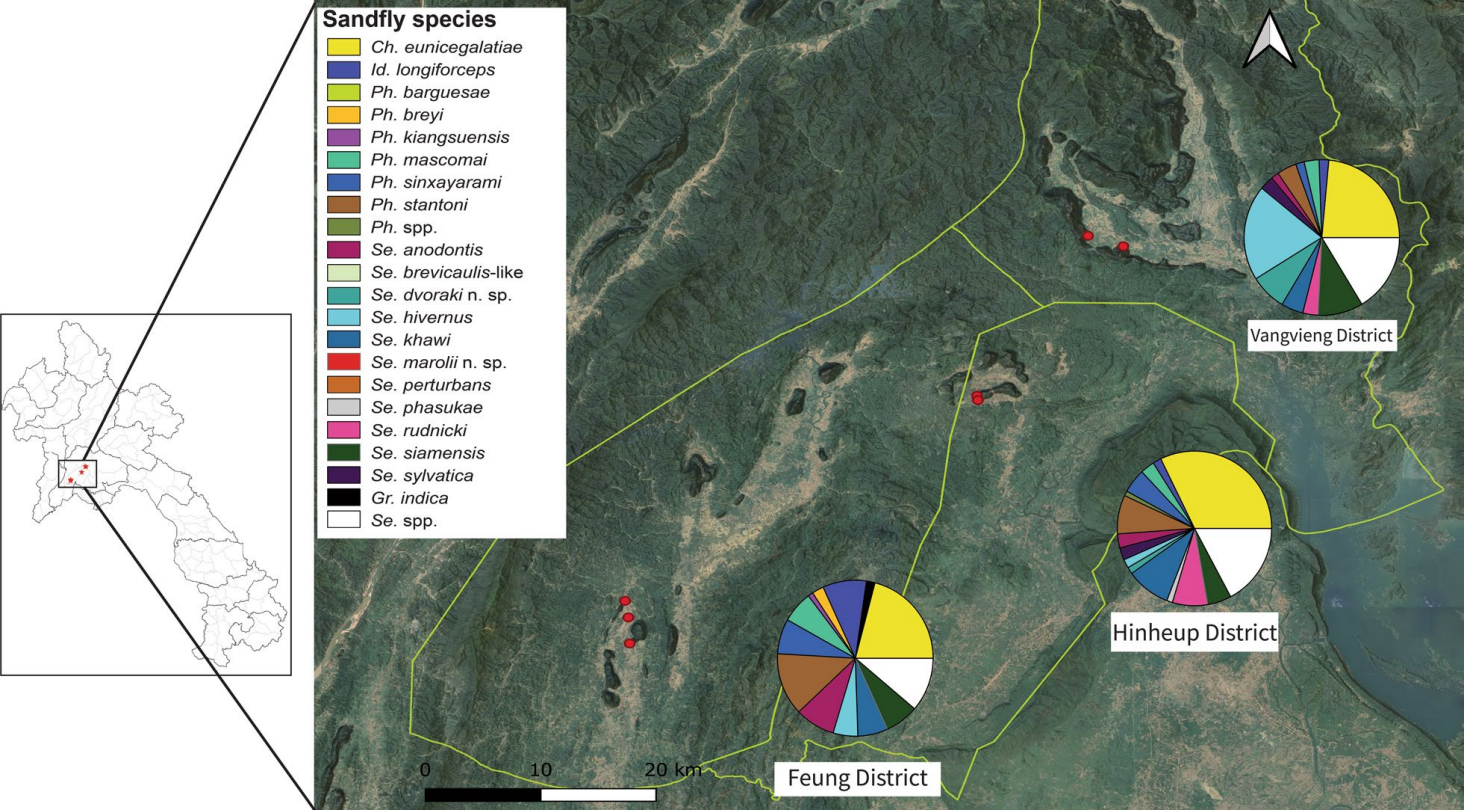
Study sites and sand fly species composition from karstic limestones in three districts of Vientiane Province. Red dots represent sampling locations

A total of seven caves in three districts with karstic limestone mountains were selected as study sites: Fueng district (18°30′N, 101°59′E), Hinheup district (18°46′N, 102°16′E), and Vangvieng district (18°58′N, 102°20′E) (Fig. 1). Among the three districts, Vangvieng is well known for cave exploration, with increasing numbers of tourists visiting each year. It is one of the top tourism destinations in Laos. In Fueng, three locations where large caves were located in each of three isolated limestones were selected for our sampling, namely, Tham Phanokkok (PNK), Tham Nam (TN), and Tham Pha (TP); in Hinheup, two caves in one isolated limestone were selected, namely, Tham Yao (TY) and Tham Phaluesy (PLS); and in Vangvieng, one large cave, Tham Nang Oau Khiem (NOK), and one touristic site in karstic areas with no large cave, namely, Angluang (AL), in a long and large limestone were selected (Additional file 1: Table S1).

### Sand fly collection

To gather baseline data on the diversity and ecology of sand flies in karstic caves and peri-karstic caves within limestone areas, we analyzed sand flies collected over a total of 202 trap-nights. Sampling occurred twice in 2019 across seven locations in three selected districts. Specifically, sand flies were collected over 72 trap-nights in Feung (21 in May and 51 in October), 40 trap-nights in Hinheup (13 in February and 27 in September), and 90 trap-nights in Vangvieng (42 in January and 48 in July). Due to limited resources and logistical constraints, the collection effort was not equal across locations. Collection times coincided with Laos’s dry season (approximately November to May) and rainy season (approximately June to October). The standard Centers for Disease Control and Prevention light traps (CDC traps) were used by setting between 04:00–06:00 p.m. and 07:00–08:00 a.m. on the next day. Sand flies were collected from three main positions—outside, at the entrance, and inside caves (as described in Additional file 1: Table S1). All insects from the traps were frozen at −20 °C for 20–30 min, after which the sand flies were sorted and counted by sex. Half of the sand fly specimens were randomly stored at −20 °C for future pathogen detection, and the remaining specimens were stored in 70–90% ethanol and then transported to the Institut Pasteur du Laos (IP-Laos) laboratory in Vientiane Capital for morphological identification.

### Specimen preparation and identification

Sand fly specimens were mounted whole-body (in toto) on slides using polyvinyl alcohol (PVA) media. To study their genetics, some sand fly specimens were prepared as follows: The head, wings, and genitalia were cut under a stereomicroscope using sterile needles. The thorax was then transferred individually into a tube labeled with the same identification number as the slide and stored at −20 °C until the molecular study was performed. The head, wings, and genitalia were mounted immediately on a slide using PVA media. All slides were morphologically identified using the dichotomous keys of Lewis and other related original description references [18, 22–29]. Males of the genus *Sergentomyia* were not identified at the species level due to the high similarity of the male morphology within this genus. Similarly, all female specimens with poor-quality slide mounting were not identified at the species level. For the descriptions of the new species, the consensual terminology of Galati et al. [30] was used.

### Molecular study

Thorax samples were homogenized for 2–5 min using a TissueLyser II system (Qiagen) with 0.5 ml of 1× phosphate-buffered saline (PBS) and Lysing Matrix E zirconium beads (MP Biomedicals). A NucleoSpin^®^8 (Macherey–Nagel) extraction kit was used to extract 100 µl of the supernatant, following the manufacturer’s protocol. All PCR amplifications were carried out in a 50 μl volume containing 5 µl of extracted DNA and 45 μl of PCR Master Mix (Promega) containing 50 pmol of each primer targeting the cytochrome b gene (*Cyt-b*), i.e., C3B-PDR (5′-CAYATTCAACCWGAATGATA-3′) and N1N-PDR (5′-GGTAYWTTGCCTCGAWTTCGWTATGA-3′), according to previously published conditions [31]. Sequencing reactions were performed using the BigDye Terminator v1.1 cycle sequencing kit (Applied Biosystems) at the IP-Laos.

### Data analysis

The sand fly density (D) was estimated as the number of sand flies collected per trap per night (trap-night). For specimens identified to species level, species richness (R) was determined as the number of sand fly species collected. All the statistical analyses were performed using R software (https://www.r-project.org/). The alpha diversity of the communities, the Shannon index (*H*) [32], was calculated, and the Kruskal‒Wallis test was subsequently used to compare the Shannon index between sites via the Rstatix package. To investigate the similarity between sites, trapping positions, and seasons in terms of species composition and density (beta diversity), the Bray‒Curtis index between sites was calculated for each trap-night. Principle coordinate analysis (PCoA) was used to display the beta diversity indices for sand flies among sites and locations. Permutational multivariate analysis of variance (PERMANOVA) was used to compare the beta diversity indices using the vegan package.

Newly generated sequences from this study were aligned with *Cyt-b* sequences available in GenBank and used for genetic analysis (Additional file 2: Table S2). Genetic distances were calculated within species and between species groups using the Tamura–Nei model. A maximum likelihood (ML) tree was constructed in MEGA 11 [33] using the substitution models selected by MODELTEST [34].

## Results

### Sand fly morphological species identification and composition

A total of 6564 sand flies were morphologically identified, of which 5038 (76.8%) were females and 1526 (23.3%) were males. The sand flies were classified into 20 species belonging to five genera: *Chinius* (one species): *Ch. eunicegalatiae*, 1555 (23.7%); *Idiophlebotomus* (one species): *Id. longiforceps*, 351 (5.4%); *Phlebotomus* (six species): *Ph. barguesae*, three (0.1%); *Ph. breyi*, 90 (1.4%); *Ph. kiangsuensis*, 51 (0.8%); *Ph. mascomai*, 307 (4.7%); *Ph. sinxayarami*, 338 (5.2%); *Ph. stantoni*, 606 (9.2%), and unclassified as mounting slides were not clear, *Phlebotomus* spp., 52 (0.8%); *Sergentomyia* (11 species): *Se. anodontis*, 337 (5.1%); *Se. siamensis*, 462 (7.0%); *Se. brevicaulis*-like, five (0.08%); *Se. hivernus*, 536 (8.1%); *Se. khawi*, 416 (6.3%); *Se. perturbans*, four (0.1%); *Se. phasukae*, 33 (0.5%); *Se. rudnicki*, 164 (2.5%); *Se. sylvatica*, 110 (1.7%); *Se. dvoraki* n. sp., 184 (2.8%); *Se. marolii* n. sp., two (0.04%); *Sergentomyia* spp., 898 (13.7%); and *Grassomyia* (one species): *Gr. indica*, 60 (0.1%); (Fig. 1 and Table 1).

**Table 1:** Sand fly species composition and abundance in three districts of Vientiane Province in this study

Species	District			Total
	Feung	Hinheup	Vangvieng	
	No. (F/M)	No. (F/M)	No. (F/M)	No. (F/M)
*Ch. eunicegalatiae*	669 (565/104)	476 (304/172)	410 (360/50)	1555 (1229/326)
*Id. longiforceps*^a^	290 (259/31)	26 (13/13)	35 (23/12)	351 (295/56)
*Ph. barguesae*^a^	2 (2/0)	1 (1/0)	–	3 (3/0)
*Ph. breyi*	78 (50/28)	6 (4/2)	6 (6/0)	90 (60/30)
*Ph. kiangsuensis*^a^	34 (28/6)	7 (7/0)	10 (10/0)	51 (45/6)
*Ph. mascomai*^a^	209 (204/5)	44 (36/8)	54 (44/10)	307 (248/23)
*Ph. sinxayarami*	232 (189/43)	75 (59/16)	31 (25/6)	338 (273/65)
*Ph. stantoni*	415 (292/123)	120 (79/41)	71 (58/13)	606 (429/177)
*Ph.* spp.	28 (19/9)	15 (12/3)	9 (4/5)	52 (35/17)
Total *Phlebotomus*	998 (784/214)	268 (198/70)	181 (10.19)	1447 (1129/318)
*Se. anodontis*^a^	266 (266/0)	43 (43/0)	28 (28/0)	337 (377/0)
*Se. brevicaulis*-like^a^	5 (5/0)	–	–	5 (5/0)
*Se. dvoraki* n. sp.^a^	31 (31/0)	21 (21/0)	132 (132/0)	184 (184/0)
*Se. hivernus*	164 (164/0)	27 (27/0)	345 (345/0)	536 (563/0)
*Se. khawi*	199 (199/0)	137 (137/0)	80 (80/0)	416 (416/0)
*Se. marolii* n. sp.^a^	2 (2/0)	–	–	2 (2/0)
*Se. perturbans*	2 (2/0)	–	2 (2/0)	4 (4/0)
*Se. phasukae*^a^	8 (8/0)	18 (18/0)	7 (7/0)	33 (33/0)
*Se. rudnicki*	–	109 (109/0)	55 (55/0)	164 (164/0)
*Se. siamensis*	224 (224/0)	74 (74/0)	164 (164/0)	462 (462/0)
*Se. sylvatica*	21 (21/0)	38 (38)	51 (51/0)	110 (110/0)
*Sergentomyia* spp.^b^	358 (24/334)	255 (36/219)	285 (12/273)	898 (898/0)
Total *Sergentomyia*	1280 (946/334)	722 (503/219)	1149 (876/273)	3151 (2325/826)
*Gr. indica*	57 (57/0)	2 (2/0)	1 (1/0)	60 (60/0)
Total	3294 (2611/683)	1494 (1020/474)	1776 (1407/369)	6564 (5038/1526)
Trap-nights	72	40	90	202
Density (average no.) of sand flies	45.75	37.35	19.73	32.5
Minimum	2	2	1	1
Maximum	202	115	102	202
Species richness	19	17	17	20
Species diversity (*H*)	2.46	2.24	2.26	2.48

^a^ New record from this study

^b^ Male specimens of genus *Sergentomyia* were identified only as *Sergentomyia* spp. and not identified to the species level due to the high similarity of the male morphology within this genus. All female specimens without good quality of slide mounting were also classified as *Sergentomyia* spp.

*F* female, *M* male

**Fig. 2:**
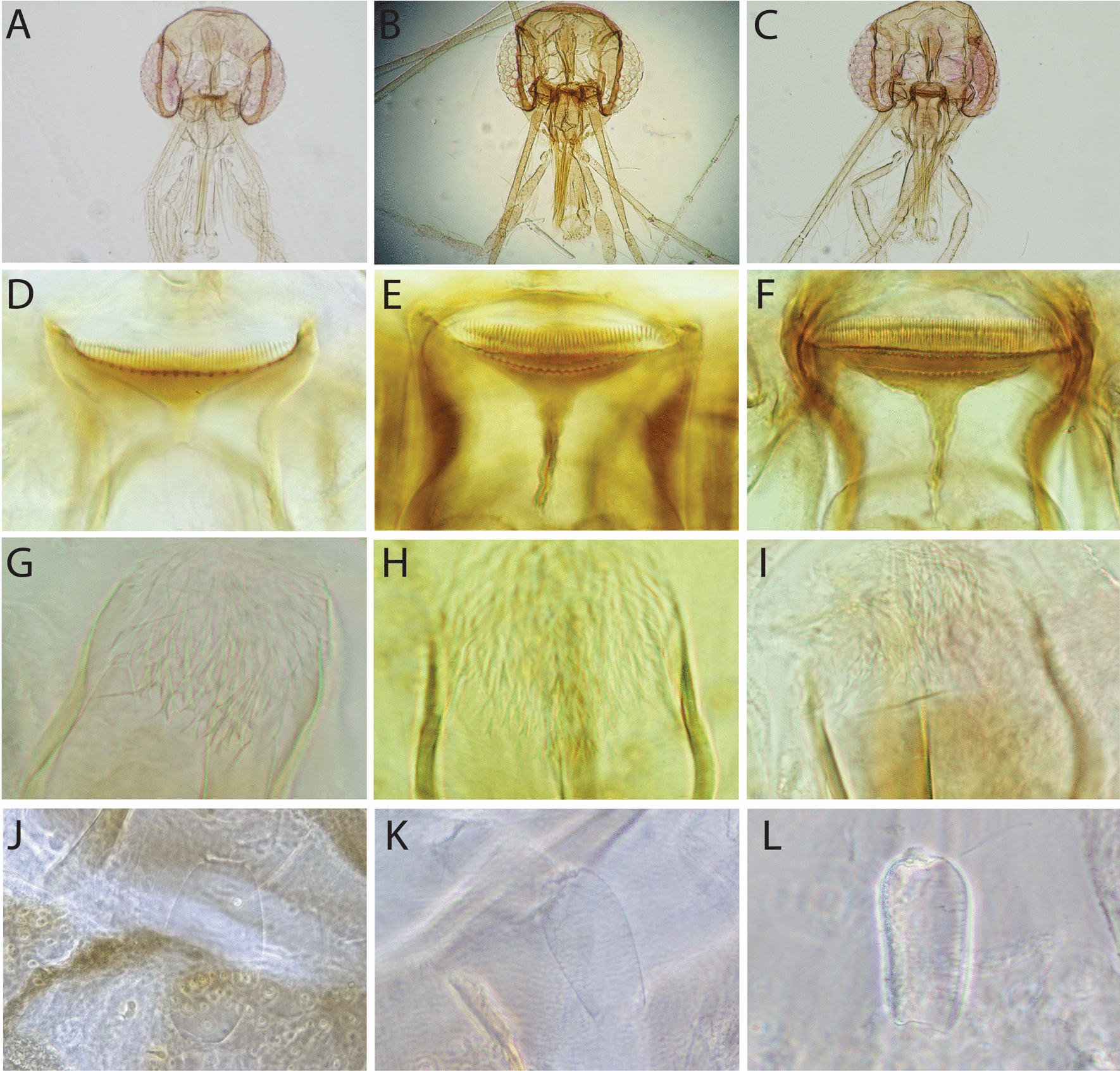
Female morphological characteristics of *Se. siamensis*, *Se. brevicaulis*-like, and *Se. rudnicki* found in this study. *Sergentomyia siamensis*: head with short flagellomere 1 (**A**), cibarium with about 54 teeth (**D**), pharyngeal teeth long (**G**), smooth and ovoid spermathecae (**J**); *Se. brevicaulis*-like teeth: head with long flagellomere 1 (**B**), cibarium with about 50 comb-like teeth (**E**), pharyngeal teeth long (**H**), oblong and wrinkled spermathecae (**K**); and *Se. rudnicki*: head with long flagellomere 1 (**C**), cibarium with about 80 comb-like teeth (**F**), pharyngeal teeth long (**I**), and oblong and wrinkled spermathecae (**L**)

**Fig. 3:**
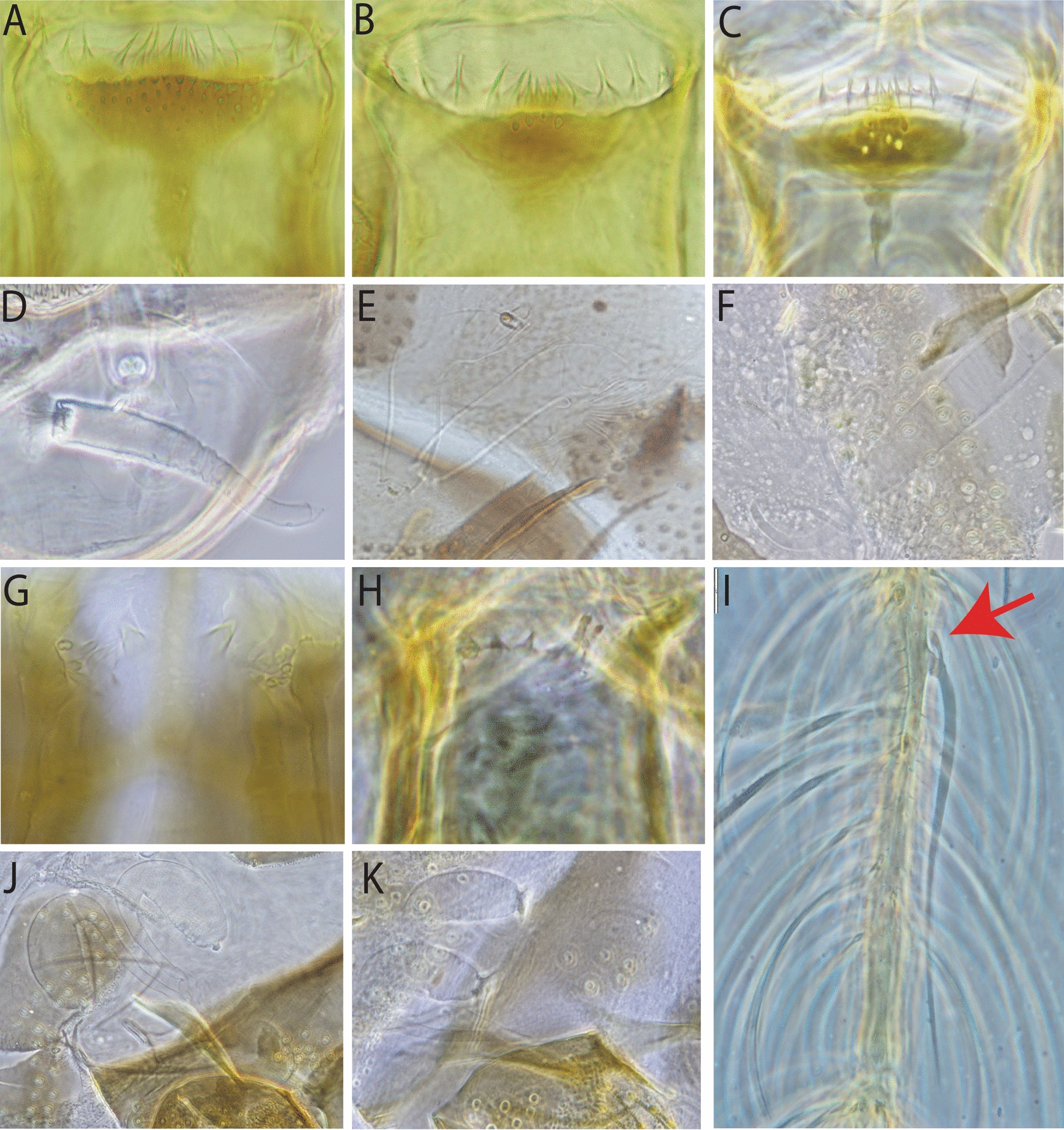
Female morphological characteristics of *Sergentomyia*. *Sergentomyia khawi*: cibarium with three rows of vertical teeth (**A**), smooth spermathecae and wide individual duct (**D**); *Se. hivenus*: cibarium (**B**) and tubular spermathecae (**E**); *Se. dvoraki* n. sp.: cibarium of paratype (**C**), spermathecae with long and narrow individual ducts (**F**); *Se. gemmea* (holotype): red arrow points to the spur of the ascoid (**I**); *Se. phasukae*: cibarial teeth, no pigment patch (**G**), spermathecae (**J**); and *Se. sylvatica*: cibarium teeth (**H**), and spermathecae (**K**)

**Fig. 4:**
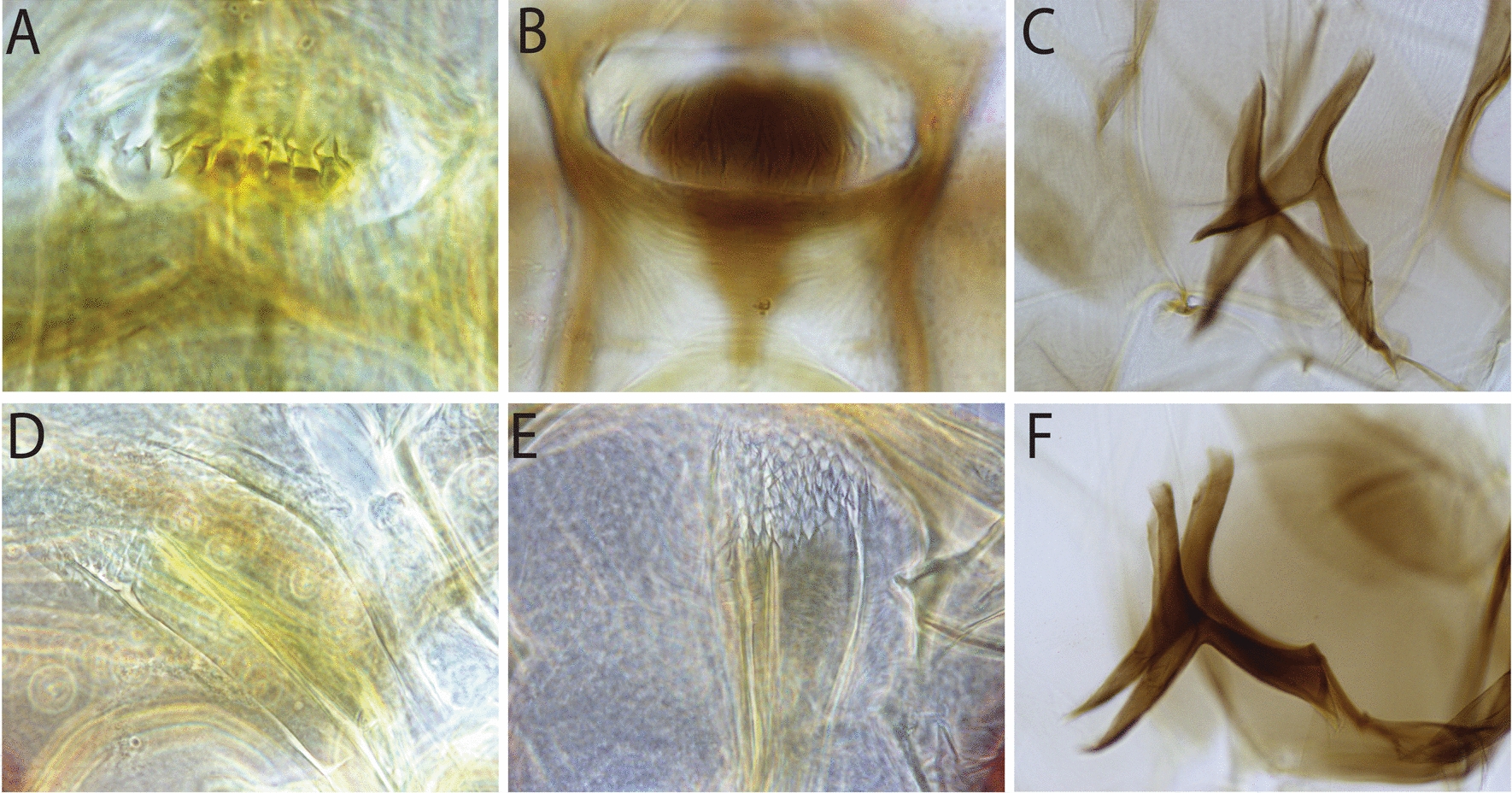
*Sergentomyia perturbans*: cibarium (**A**), pharynx unarmed (**D**), and mesofurca strongly pigmented (**F**); and *Se. marolii* n. sp.: cibarium (**B**), pharynx armed with teeth (**E**), and mesofurca strongly pigmented (**C**)

Regarding the taxonomy of sand flies within the genus *Sergentomyia* in this study, specimens were classified as *Se. siamensis* based on the following characters: around 54 comb-like teeth (posterior teeth) with two rows of anterior teeth (fore-teeth) of about 22 and six on the cibarium, short antenna flagellomer1 (f1) less than 190 µm, and its length about half of the length of proboscis, ovoid and smooth spermathecae (Fig. 2A, D, G, and J); as *Se. brevicaulis*-like for those that have long antennae f1, extending beyond the tip of the proboscis with about 50 posterior comb-like teeth on cibarium (Fig. 2B, E, H, and K); and as *Se. rudnicki* for those that have long antennae f1, longer than 250 µm, extending beyond the tip of the proboscis, with about 80 posterior comb-like teeth on the cibarium and two rows of anterior teeth of more than 30 on each row, with oblong and annulate spermathecae (Fig. 2C, F, I, and K). Main morphological characteristics, cibarial teeth, and spermathecae for the diagnosis of *Se. khawi*, *Se. hivernus*, *Se. dvoraki* n. sp., *Se. marolii* n. sp., *Se. phasukae*, *Se. perturbans*, and *Se. sylvatica* are shown in Figs. 3 and 4. Descriptions of *Se. dvoraki* n. sp. and *Se. marolii* n. sp. are provided below. Comments on the taxonomy of the sand flies in this study are provided in the Discussion section.

### Phylogenetic analysis and genetic distance

**Fig. 5:**
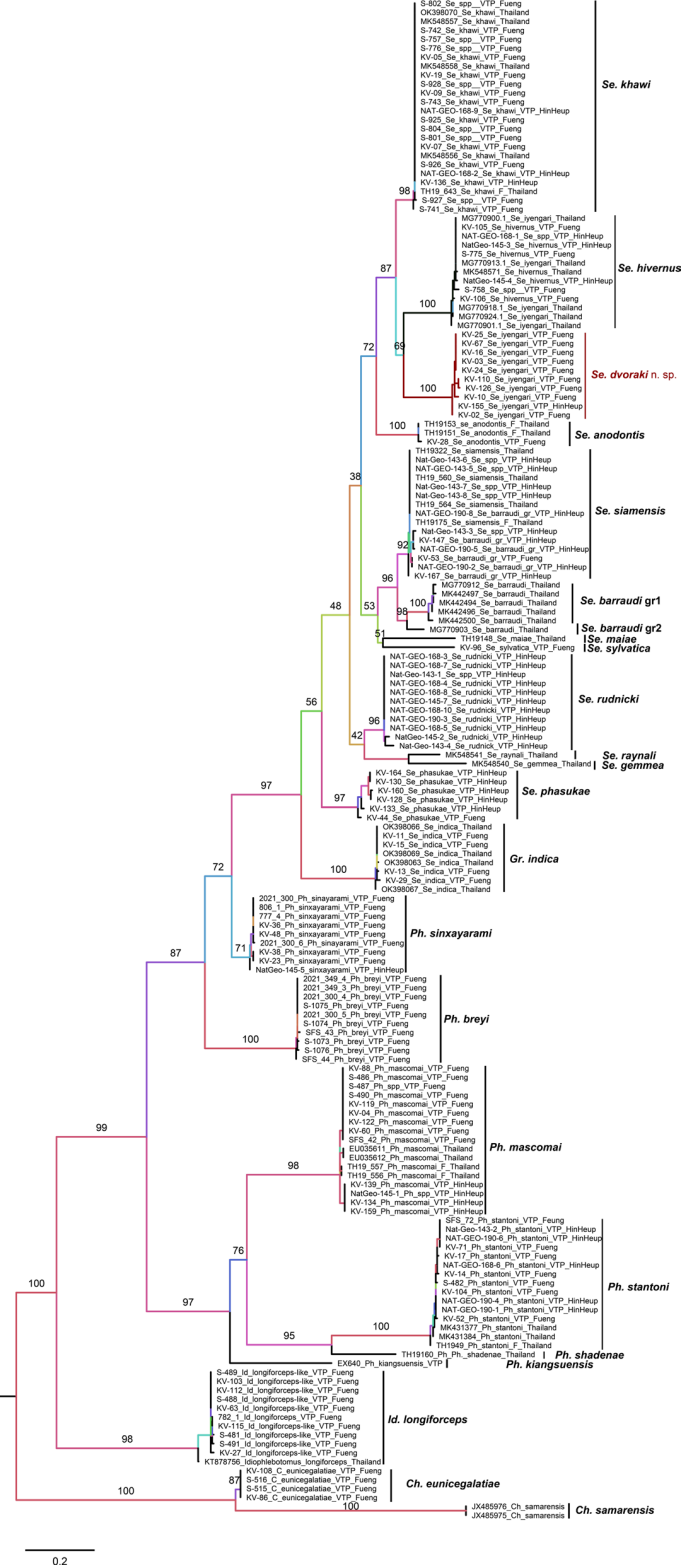
Maximum likelihood tree constructed from cytochrome b gene using the Tamura–Nei model

A total of 129 samples were successfully amplified, and the following *Cyt-b* sequences were obtained: *Ch. eunicegalatiae* (four specimens), *Id. longiforceps* (10), *Ph. breyi* (10), *Ph. kiangsuensis* (one), *Ph. mascomai* (13), *Ph. sinxayarami* (nine), *Ph. stantoni* (12), *Se. anodontis* (one), *Se. hivernus* (seven), *Se. dvoraki* n. sp. (10), *Se. khawi* (19), *Se. phasukae* (six), *Se. rudnicki* (11), *Se. siamensis* (11), *Se. sylvatica* (one), and *Gr. indica* (four). We did not sequence *Cyt-b* of other rare species, such as *Ph. barguesae*, *Se. brevicaulis*-like, *Se. perturbans*, and *Se. marolii* n. sp., because the whole bodies of all these samples were mounted (in toto). Together with *Cyt-b* sequences available from Thailand, a total of 170 sequences were analyzed (Additional file 2: Table S2). A phylogenetic ML tree constructed using the Tamura–Nei model, which was the best-fit nucleotide substitution model for this study, showed that all species used in these analyses formed monophyletic clades, in particular for the *Chinius*, *Grassomyia*, *Idiophlebotomus*, and *Phlebotomus* genera. Interestingly, for the genus *Sergentomyia*, all the *Se. siamensis* from this study formed a subclade with two other subclades of the *Se. barraudi* group from Thailand, labeled *Se. barraudi* group 1 (MG770912, MK442497, MK442494, MK442496, and MK442500) and group 2 (MG770903) (Fig. 5). The mean pairwise distance between *Se. siamensis* and *Se. barraudi* group 1 and group 2 was 0.08 and 0.07, respectively, and between *Se. rudnicki* and *Se. siamensis* it was 0.10. Genetic analysis supported our morphological observation on the most closely related species. The mean pairwise distance between *Se. dvoraki* n. sp. and *Se. khawi* was 0.12, and between *Se. dvoraki* n. sp. and *Se. hivernus* it was 0.13. The mean pairwise distances within groups ranged from 0 to 0.018, and between group species it ranged from 0.07 to 0.41. (Further details on the pairwise mean distances between and within species are also provided in Additional file 3: Table S3.)

### Sand fly diversity and species assemblages across sampling sites and cave locations

Among the sand flies identified, 3294 (50.18%) were collected from Feung (72 trap-nights, density (D) = 45.75 [Min–Max: 2–202]), 1494 (22.76%) from Hinheup (40 trap-nights, D = 37.35 [Min–Max: 2–115]), and 1776 (27.06%) from Vangvieng (90 trap-nights, D = 19.73 [Min—Max: 1–102]) (Table 1). For the diversity analysis, species accumulation curves were plotted by site, season, and position of trapping to estimate whether they could be representative of total sand fly identification. Species accumulation reached a plateau in this study, especially during the rainy season, with a high overall Shannon diversity index *H* in the three districts (Additional file 4: Fig. S1). In Feung district, the species richness (R) = 19, Shannon index (*H*) = 2.46, and the most abundant species were *Ch. eunicegalatiae* (20.31%), *Id. longiforceps* (8.80%), *Ph. stantoni* (12.60%), and *Se. anodontis* (8.08%); in Hinheup district, R = 17 species, *H* = 2.24, and the most abundant species were *Ch. eunicegalatiae* (31.86%), *Ph. stantoni* (8.03%), *Se. khawi* (9.17%), and *Se. rudnicki* (7.3%); and in Vangvieng district, R = 17 species, *H* = 2.26, and the most abundant species were *Ch. eunicegalatiae* (23.09%), *Se. hivernus* (19.43%), and *Se. siamensis* (9.23%) (Table 1). Regarding the species assemblages between districts, the Bray‒Curtis index values for all the trap-nights differed significantly across the three sampling districts, especially between Vangvieng and Hinheup and between Vangvieng and Feung, but there was no significant difference between Hinheup and Feung (Additional file 5: Fig. S2).

At the cave location level, we observed that sand fly density was high in all cave areas (D > 30 sand flies/trap-night), except in NOK cave in the Vangvieng district, where only eight sand flies per trap-night were found. The species richness ranged from 14 to 18, of which the lowest was in the NOK cave in Vangvieng district (Additional file 6: Table S4). Species assemblages differed greatly among our seven sampling locations (Additional file 5: Fig. S2).

Different density and *H* index values were also observed among the trapping positions. For sand flies outside caves, R = 20, D = 34.29, and *H* = 2.50; and for cave entrances, R = 18, D = 40.28, and *H* = 2.41. These values were significantly greater than those for inside caves: R = 16, D = 22.57, and *H* = 2.13. Among all the sand fly species, *Ch. eunicegalatiae* and *Ph. stantoni* were the most abundant species inside, at the entrance, and outside caves (Additional file 7: Table S5).

From two collections, one in the dry season and another in the rainy season, the sand fly density was high in both the rainy and dry seasons in Fueng and Hinheup. However, it was low in the dry season in Vangvieng (D = 6.29) (Additional file 8: Table S6).

**Descriptions of new species**

**Family **Psychodidae Newman, 1834

**Genus ***Sergentomyia* França and & Parrot, 1920

**Subgenus** ungrouped awaiting a *Sergentomyia* taxonomic revision.

**Fig. 6:**
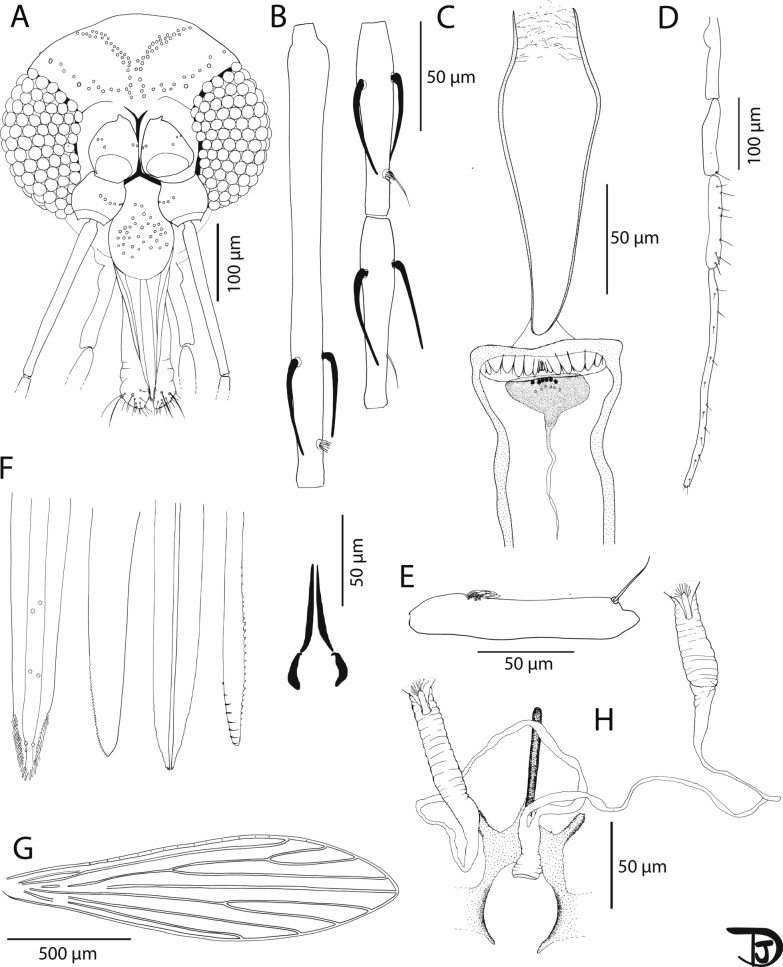
*Sergentomyia dvoraki* n. sp. female. **A** Head (paratype LAOS#251-2); **B** flagellomeres 1, 2, and 3 (= AIII, AIV, and AV) (paratype LAOS#251-2); **C** pharynx and cibarium (holotype LAOS#251-3); **D** palp (paratype LAOS#251-2); **E** third segment of the palp (P3) (paratype LAOS#251-2); **F** mouth parts (labrum, hypopharynx, mandible, maxilla [paratype LAOS#251-2], and labial furca [paratype LAOS#251-2] from left to right); **G** wing (holotype LAOS#251-3); **H** furca and spermathecae (holotype LAOS#251-3)

*Sergentomyia dvoraki* n. sp. Randrianambinintsoa, Vongphayloth and Depaquit, 2024 (Fig. 6 and Table 2).

**Table 2:** *Sergentomyia dvoraki* n. sp. female measurements (in µm)

Variables		No. of samples	Mean	Minimum	Maximum	SD
Head	Length	8	339.49	314.1	356.42	14.81
	Width	8	345.02	310.33	382.35	23.46
	Clypeus	8	112.1	101.21	121.71	8.35
	Labrum	8	173.17	160.93	189.05	9.78
Flagellomeres	f1	8	205.83	181.82	224.03	15.59
	f2	8	91.67	87.55	96.46	2.85
	f3	8	92.6	85.19	104.96	6.2
Palpi	p1	8	30.3	23.7	41.91	5.32
	p2	8	76.93	71.84	87.52	4.96
	p3	8	110.29	99.89	125.88	7.24
	p4	8	122.54	115.77	140.4	7.98
	p5	7	247.41	186.73	296.56	42.8
Cibarium	Anterior teeth	8	6	2	12	3.36
	Posterior teeth	8	15	14	16	0.93
Wing	Length	8	1555.62	1516.44	1587.58	28.07
	Width	8	421.27	401.12	443.44	13.17
	α (r2)	8	257.49	218.25	300.25	34.24
	ε (r3)	8	374.99	342.37	425.22	31.38
	θ (r4)	8	816.4	782.19	850.42	24.21
	r5	8	1097.74	1049.31	1138.97	25.46
	β (r2 + r3)	8	329.49	287.95	374.36	28.37
	δ (r2 + 3 to r1)	8	122.5	75.46	180.31	36.36
	γ (r2 + 3 + 4)	8	252.82	237.57	281.24	13.69
	π (r2 + 3 to m1 + 2)	8	142.79	81.01	215.29	42.36
	α/β (r2/r2 + 3)	8	0.79	0.58	1.04	0.16
	Width/γ	8	1.67	1.51	1.87	0.11
Spermathecae^a^	Body length	1	69	–	–	–
	Body width	1	20	–	–	–
	Common duct	1	29	–	–	–
	Individual ducts	1	255	–	–	–

^a^Spermathecae from the holotype

***Type locality*** Pha Nok Kok cave (18°30′N, 101°59′E), Feung district, Vientiane Province, Laos.

***Type specimens*** The female holotype (voucher LAOS-251-3) of *Se. dvoraki* n. sp. and two female paratypes (voucher LAOS-251-1, LAOS-251-2) have been deposited at the terrestrial arthropod collection of the Muséum national d’Histoire naturelle (MNHN, Paris) under inventory numbers MNHN-ED-115949–MNHN-ED-11951.

***ZooBank registration*** To comply with the regulations set out in Article 8.5 of the amended 2012 version of the International Code of Zoological Nomenclature (ICZN) [35], details of the new species have been submitted to ZooBank. The Life Science Identifier (LSID) of the article is urn:lsid:zoobank.org:pub:AB876763-B1CC-4414-A45E-24247A3F33CD. The LSID for the new species *Se. dvoraki* is urn:lsid:zoobank.org:act:566AB258-2548-41BF-ADE1-1878E0730B85.

***Etymology*** The species *Se. dvoraki* n. sp. is dedicated to our colleague Vit Dvořák for his important contribution to research in the field of phlebotomine sand flies and leishmaniasis.

**Description**

Measurements and counts indicated are those of the holotype (voucher LAOS-251).

**Female**: Head: occiput with two narrow lines of well-individualized setae. Clypeus 115 µm long and 86 µm wide with 42 setae randomly distributed. Eyes 177 µm long, 97 µm wide, with about 90 facets. Interocular sutures incomplete. Interantennal sutures do not reach the interocular sutures. Flagellomere 1 longer than f2 + f3. Presence of two ascoids from f1 to f13. Absence on f14. Ascoidal formula: 2/f3–f13 with long ascoids, not reaching the next article. One papilla on f1 and f2. Absence of papilla from f3 to f9, two papillae on f10 and f11 (one basal and one apical), four on f12 and 13 (two basal and two apical), and four basal papillae on f14. No simple setae on f1 to f4, one on f5, f6, f8, and f9; two on f7; 20–25 on f14. Palpal formula: 1, 2, 3, 4, 5. Presence of a group of about 10 club-like Newstead’s sensillae implanted proximally on the third palpal segment. Presence of one distal simple seta on p3, eight on p4, and 16 on p5 on the paratype LAOS#251-2 (the p5 of the holotype is broken). Labrum 164 µm long. About 20 distal teeth on each side. Hypopharynx with around 15 distal undulations on each side of the salivary canal. Mandibles with a little more than 30 small lateral teeth. Maxillary lacinia exhibits eight external and about 25 internal teeth. Labial furca incomplete. Cibarium armed with 18 posterior teeth, and two central rows of anterior teeth: six bigger and six smaller. Sclerotized area rounded, not reaching the lateral side of the cibarium. Sclerotized arc not observed. Thorax: dark brown sclerites. Mesonotum: absence of post-alar seta. Absence of proepimeral; absence of upper and lower anapisternal seta; absence of anepimeral seta; absence of metaepisternal seta; absence of metaepimeral seta; presence of some fine setae on the anterior region of the katepisternum. Metafurca with long and separated vertical and horizontal arms. Two cervical sensillae on each side. One ventro-cervical sensilla. Wings: length = 1536 μm; width = 401 μm. r5 = 1049 μm, α (r2) = 224 μm, β (r2 + 3) = 324 μm, δ (r2 + 3-r1) = 80 μm, γ (r2 + 3 + 4) = 250 μm, ε (r3) = 343 μm, θ (r4) = 782 μm, π = 157 μm. Width/γ = 1.60, α (r2)/β (r2 + 3) = 0.69. Legs: anterior leg: coxa = 254 µm; femur = 510 µm; tibia = 492 µm; tarsomere i = 274 µm; sum of tii, tiii, tiv, tv = 455 µm.

Median leg: coxa = 226 µm; femur = 555 µm; tibia = 597 µm; tarsomere i = 322 µm; sum of tii, tiii, tiv, tv = not observed. Posterior leg: coxa = 270 µm; femur = 648 µm; tibia = 748 µm; tarsomere i = 387 µm; sum of tii, tiii, tiv, tv = 564 µm. Abdomen: setae randomly distributed on the first tergite. Genitalia: narrow wrinkled spermathecae, with thin walls. Terminal knob deeply embedded in the capsule. Short smooth common duct. Long smooth individual ducts. Genital furca with two thin and well-developed lateral process.

**Male**: Unknown.

**Family **Psychodidae Newman, 1834

**Genus ***Sergentomyia* França and & Parrot, 1920

**Subgenus** ungrouped awaiting a *Sergentomyia* taxonomic revision.

**Fig. 7:**
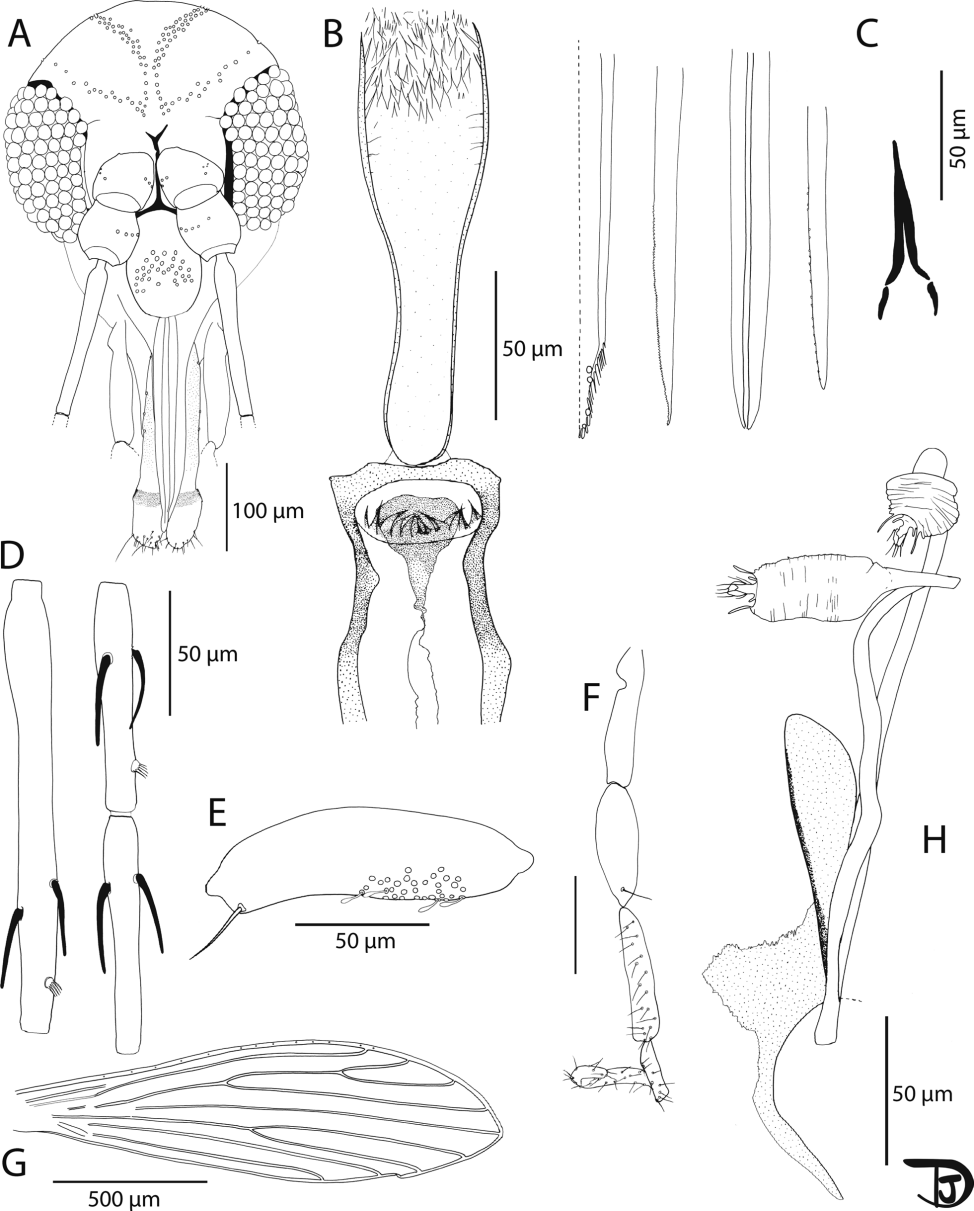
*Sergentomyia marolii* n. sp. female. **A** Head (paratype S-1610-2); **B** pharynx and cibarium (holotype S-2553-4); **C** mouth parts (labrum, hypopharynx, mandible, maxilla, and labial furca from left to right) (paratype S-1610-2); **D** flagellomeres 1, 2, and 3 (= AIII, AIV, and AV) (holotype S-2553-4); **E** third segment of the palp (P3) (holotype S-2553-4); **F** palp (paratype S-1610-2); **G** wing (holotype S-2553-4); and **H** furca and spermathecae (paratype)

*Sergentomyia marolii* n. sp. Vongphayloth, Randrianambinintsoa and Depaquit, 2024 (Fig. 7A–H and Table 3).

**Table 3:** *Sergentomyia marolii* n. sp. female measurements (in µm)

Variables		Holotype (S-2553-4)	Paratype (S-1610-2)
Head	Length	313.89	342.95
	Width	297.75	322.02
	Clypeus	112.33	114.23
	Labrum	228.45	256
Flagellomeres	f1	177.98	177.67
	f2	90.4	86.7
	f3	94.07	92.6
Palpi	p1	32.36	34.5
	p2	95.08	97.13
	p3	107.71	111.85
	p4	123.59	128.97
	p5	204.92	–
Cibarium	Anterior teeth	11	11
	Posterior teeth	Absence	Absence
Wing	Length	1582.03	–
	Width	435.54	–
	α (r2)	203.13	–
	ε (r3)	362.15	–
	θ (r4)	815.4	–
	r5	1151.84	–
	β (r2 + r3)	355.32	–
	δ (r2 + 3 to r1)	76.79	–
	γ (r2 + 3 + 4)	289.47	–
	π (r2 + 3 to m1 + 2)	80.91	–
	α/β (r2/r2 + 3)		–
	Width/γ		–
Spermathecae	Body length	35	30
	Body width	18	18
	Common duct	–	Difficult to observe
	Individual ducts	–	Approximately 160

***Type locality*** Pha Nok Kok cave (18°30′N, 101°59′E), Feung district, Vientiane Province, Laos.

***Type specimens*** The holotype female (voucher S-2553-4) and one female paratype (voucher S-1610-2) of *Se. marolii* n. sp. have been deposited at the terrestrial arthropod collection of the Muséum national d’Histoire naturelle (MNHN, Paris) under inventory numbers MNHN-ED-115952 and MNHN-ED-115953.

***ZooBank registration*** To comply with the regulations set out in Article 8.5 of the amended 2012 version of the International Code of Zoological Nomenclature (ICZN) [35], details of the new species have been submitted to ZooBank. The Life Science Identifier (LSID) of the article is urn:lsid:zoobank.org:pub:AB876763-B1CC-4414-A45E-24247A3F33CD. The LSID for the new species *Se. marolii* is urn:lsid:zoobank.org:act:FB152938-A7FD-4B14-90F7-73485F508B94.

***Etymology*** The species *Se. marolii* n. sp. is dedicated to our colleague Michele Maroli for his important contribution to research in the field of phlebotomine sand flies and leishmaniasis, and in hosting the first International Symposium on Phlebotomine Sandflies (ISOPS).

**Description**

Measurements indicated are those of the holotype (voucher S-2553-4.)

**Female**: Head: occiput with two narrow lines of well-individualized setae. Clypeus 112 µm long and 77 µm wide with 34 setae randomly distributed. Eyes 167 µm long, 93 µm wide, with about 80 facets. Interocular sutures incomplete. Interantennal sutures do not reach the interocular sutures. Flagellomere 1 shorter than f2 + f3. Presence of two ascoids from f1 to f13 and absence on f14. Ascoidal formula: 2/f3-f13 with ascoids not reaching the next article. One papilla on f1 and f2. Absence of papilla from f3 to f9. One on f10 and f11, three from f12–f14. No simple setae from f1 to f11, one on f12, two on f13 and less than 25 on f14. Palpal formula: 1, 2, 3, 4, 5. Presence of a group of about 30 club-like Newstead’s sensillae implanted on the proximal half on the third palpal segment. Presence of one distal simple seta on p3, 17 on p4, and about 30 on p5. Labrum 228 µm long with about 15 lateral teeth. Smooth lateral sides of the hypopharynx. Thin maxillary lacinia with about 90 lateral little teeth. Labial furca complete. Cibarium armed with 11 big teeth organized along a very slightly curved line. No denticles (anterior teeth). Sclerotized area strongly pigmented, thick and narrow (not reaching the lateral walls of the cibarium). Pharynx strongly armed with pointed translucent teeth oriented backwards, on its posterior quarter. Thorax: light brown sclerites. Mesonotum: absence of post-alar seta. Proepimeral setae non-observed; absence of upper anepisiternal seta; absence of lower anepisiternal setae; absence of anepimeral seta; absence of metaepisternal seta; absence of metaepimeral seta; presence of a tuft of long and fine setae on the anterior region of the katepisternum. Metafurca with separated long vertical and short horizontal arms. Mesofurca dark brown.

Cervix with two cervical sensillae on each side. Two ventro-cervical sensillae. Wings: length = 1582 μm; width = 435 μm. r5 = 1151 μm, α (r2) = 203 μm, β (r2 + 3) = 355 μm, δ (r2 + 3-r1) = 76 μm, γ (r2 + 3 + 4) = 289 μm, ε (r3) = 362 μm, θ (r4) = 815 μm, π = 80 μm. Width/γ = 1.5, α (r2)/β (r2 + 3) = 0.57. Legs (on S-2553-4): anterior leg: coxa = 247 µm; femur = 566 µm; tibia = 604 µm; tarsomere i = 339 µm; sum of tii, tiii, tiv, tv = 516 µm. Median leg: coxa = 263 µm; femur = 617 µm; tibia = 763 µm; tarsomere i = 427 µm; sum of tii, tiii, tiv, tv = 563 µm. Posterior leg: coxa = 273 µm; femur = 627 µm; tibia = 967 µm; tarsomere i = 485 µm; sum of tii, tiii, tiv, tv = 617 µm. Abdomen: setae randomly distributed on the first tergite. Genitalia: short, wrinkled oblong spermathecae, with thin walls. Terminal knob not embedded in the capsule, with setae around the knob and lateral structures surrounding it. On paratype S-1610-2: smooth individual ducts about 160 µm, common duct was not observed. Genital furca with a wide handle.

**Male**: Unknown.

## Discussion

This is the first inventory of the sand fly fauna in karstic limestone areas known in Laos. Before these data, a total of 15 species were reported from Laos, including (1) *Ch. eunicegalatiae*, (2) *Gr. indica* [as *Phlebotomus* (*Se.*) *squamipleuris*], (3), *Ph. argentipes*, (4) *Ph. breyi*, (5) *Ph. sinxayarami*, (6) *Ph. shadenae*, (7) *Ph. stantoni*, (8) *Se. bailyi*, (9) *Se. barraudi*, (10) *Se. gemmea*, (11) *Se. hivernus*, (12) *Se. iyengari* (may correspond to *Se. dvoraki* n. sp.), (13) *Se. khawi*, (14) *Se. perturbans*, and (15) *Se. sylvatica* [18, 22–25]. Here, we added 10 more species found in Laos, including (1) *Id. longiforceps*, (2) *Ph. barguesae*, (3) *Ph. kiangsuensis*, (4) *Ph. mascomai*, (5) *Se. anodontis*, (6) *Se. brevicaulis*-like, (7) *Se. dvoraki* n. sp., (8) *Se. marolii* n. sp., (9) *Se. phasukae*, and (10) *Se. rudnicki*. Dichotomous keys for females and males found in this study are provided in Tables 4 and 5. The updated list of the sand fly species of Laos includes now 25 species.

**Table 4:** Dichotomous key for females found in the karst area of Vientiane

1	Five radial veins on the wing	2
	Four radial veins on the wing (fusion of r2 + r3)	*Ch. eunicegalatiae*
2	The longest palpal segment is the distal one (p5)	3
	The longest palpal segment is not the distal one (p5)	*Id. longiforceps*
3	Abdominal tergites 2–6 with erect hairs	4
	Abdominal tergites 2–6 with recumbent hairs	9
4	Non-pigmented pharyngeal armature	5
	Pigmented pharyngeal armature	6
5	Fourth palpal segments > 130 µm, terminal knob of the spermatheca carried by a long and narrow neck	*Ph. breyi*
	Fourth palpal segments < 120 µm, terminal knob of the spermatheca carried by short and wide neck	*Ph. sinxayarami*
6	Presence of a thick and long common spermathecal duct	*Ph. stantoni*
	Absence of a thick and long common spermathecal duct	7
7	Smooth spermathecae	*Ph. barguesae*
	Annealed spermathecae	8
8	Rounded distal ring. Limit between spermathecae and ducts difficult to observe	*Ph. mascomai*
	Rectangular distal ring. Limit between spermathecae and ducts easy to observe	*Ph. kiangsuensis*
9	Absence of a cibarial sclerotized area	*Se. phasukae*
	Presence of a cibarial sclerotized area	11
10	Little sclerotized area	*Se. sylvatica*
	Well-developed sclerotized area	11
11	Two ascoids on flagellomere 1	12
	One ascoid on flagellomere 1	*Gr. indica*
12	Presence of several cibarial teeth	13
	Presence of one big V-shaped cibarial tooth	*Se. anodontis*
13	Presence of numerous comb-like cibarial teeth	14
	Presence of pointed cibarial teeth	16
14	Bifurcated proximal part of the sclerotized area	*Se. siamensis*
	Non-furcated proximal part of the sclerotized area	15
15	More than 75 cibarial teeth	*Se. rudnicki*
	From 45 to 65 cibarial teeth	*Se. brevicaulis* group
16	Cibarium with less than 10 vertical teeth	*Se. perturbans s. l*
	Cibarium with more than 10 vertical teeth	17
17	Pharynx armed with pointed teeth oriented backwards, wrinkled spermathecae	*Se. dvoraki* n. sp.
	Pharynx armed with faint ridges	18
18	Cibarium without vertical teeth	*Se. marolii* n. sp.
	Cibarium with vertical teeth	19
19	Cibarium with two or more rows of vertical teeth occupying all the cibarial width, spermathecae are not tubular with limits between the body and duct	*Se. khawi*
	Cibarium with one or two rows with a few vertical teeth in the center of the cibarium, spermathecae are tubular without limits between the body and duct	*Se. hivernus*

**Table 5:** Dichotomous key for males found in the karst area of Vientiane

1	Five radial veins on the wing	2
	Four radial veins on the wing (fusion of r2 + r3)	*Ch. eunicegalatiae*
2	Absence of abdominal rods surrounding the aedeagal ducts	3
	Presence of abdominal rods surrounding the aedeagal ducts	*Id. longiforceps*
3	Five spines on the gonostyle	4
	Four spines on the gonostyle	8
4	Simple paramere	5
	Tri-lobed paramere	6
5	Aedeagal ducts > 500 µm	*Ph. breyi*
	Aedeagal ducts < 500 µm	*Ph. sinxayarami*
6	One ascoid on flagellomeres 2 and 3	*Ph. kiangsuensis*
	Two ascoids on flagellomeres 2 and 3	7
7	Parameral sheath slender on all their length with a round top, aedeagal ducts/pump ratio > 4	*Ph. mascomai*
	Thick parameral sheath with a blunt-end top, aedeagal ducts/pump ratio < 4	*Ph. barguesae*
8	Simple paramere	9
	Tri-lobed paramere	*Ph. stantoni*
9	Two ascoids on flagellomere 1	*Sergentomyia* spp*.*
	One ascoid on flagellomere 1	*Gr. indica*

The present study also highlights the high abundance and diversity of sand flies in all cave locations and districts in karstic areas of Vientiane Province. Heterogeneous species composition according to caves, trapping positions, and seasons was also observed. Similar results of high density and diversity, especially in karstic cave areas, and seasonal variety were also observed in Thailand, where more than 27 species of sand flies have been identified [36–39]. Differences in sand fly diversity and species composition between different specific environments suitable for sand flies were also observed elsewhere in SE Asia, Africa, and South America [40–42]. The lowest sand fly density found in this study was at NOK cave in Vangvieng district, which could be explained by the fact that this large cave is located higher from the ground than other caves are, and the cave floor might not be suitable for the development of sand fly larvae due to the substrate with low levels of organic materials. Although many sand fly species are widely distributed among SE Asian countries, many of them might be restricted to a specific habitat within an area. However, our study has limitations. The sampling method used may not have been optimal. The traps and their placement might not have captured all species, possibly underestimating the true diversity and richness. Differences in trap effectiveness, weather conditions, and timing during sampling can also cause biases in the data. To address these limitations, future studies should use more standardized and extensive sampling methods, including multiple techniques and repeated sampling over time. A more detailed analysis of beta diversity could also offer better insights into the ecological relationships and processes affecting sand fly populations in different districts. More intensive studies of sand flies in Laos, as well as other countries in the region, will provide a clearer picture of sand fly diversity and ecology.

### Taxonomic and biological comments on the species recorded in Laos

#### Genus *Chinius*

***Chinius eunicegalatiae*** was originally described from a cave in Vangvieng district [23], where this area was also selected for collection in this study. This species, as well as the genus *Chinius*, was considered exclusively cave-dwelling [23]. Interestingly, we observed in the present study that *Ch. eunicegalatiae* was not restricted only to caves, taking into consideration we also caught it inside caves or at the entrance of the caves, as well as outside caves. In the future, new studies placing traps at successive distances (at least 5 m) from the cave entrance would make it possible to learn more about the habitat of this species. It seems that this species is distributed only in the limestone areas of Laos. Until now, it has not been reported in other countries in this region. *Chinius eunicegalatiae* shares with *Ch. barbazani* the lack of r2 veins on the wing in both genders. They differ mostly in males by the position of the most proximal spine of the gonostyle, by the beveled or truncated end of the aedeagal ducts, and by the sperm pump/aedeagal ducts ratio. The females differ by the diameter of the spermathecae and by the lengths of (spermathecae + individual duct)/length of common duct ratio.

#### Genus *Idiophlebotomus*

***Idiophlebotomus longiforceps*** had never been recorded in Laos before this study. It was found inside caves or at the entrance of the caves, as well as outside caves. Our morphological characteristics and genetic analysis revealed that the Laotian specimens were similar to those recorded in Thailand and identified as *Id. longiforceps* [43]. However, this species was described from China several decades ago, in the genus *Sergentomyia* [44], and the differential characters within the genus *Idiophlebotomus* are not precisely detailed. Lewis, in his review of sand flies of the Eastern Region [18], did not report this species from SE Asia, but it has recently been reported from Vietnam [42] and Thailand [27, 45]. A revision of this genus with more specimens and with molecular studies together with comparisons to type specimens is still warranted. But here, we identified this species based on morphological characteristics similar to those of male genitalia according to the original description and identification key provided by Loyer et al. [43]. The male has a slender and rounded aedeagus, with three spines on the style: one apical, one proximal that is inserted at 2/3 of the style, and the last one is inserted near the proximal one. The coxite from the original description of this species was broader than that of other known *Idiophlebotomus* species in the revision of Lewis [18].

#### Genus* Phlebotomus*

Before the studies carried out by our laboratories, only two species were reported from Laos by Quate [24]: *Ph. argentipes* from Luangphabang and *Ph. stantoni* from Vientiane. We recently recorded and described three new species in Laos, including *Ph. breyi*, *Ph. shadenae*, and *Ph. sinxayarami* [25, 27]. In the present study, we also report *Ph. barguesae*, *Ph. kiangsuensis*, and *Ph. mascomai* in Laos. *Phlebotomus mascomai*, *Ph. sinxayarami*, and *Ph. stantoni* were the most abundant among this genus and were found inside, at the entrance, and outside caves. It is likely that these species could seek a host from both inside and outside caves. It was already observed that *Ph. kiangsuensis* can bite humans and attack bats [18]. For an accurate identification of this species, a comparison with Chinese topotypes is necessary to obtain both molecular and morphological characters. *Phlebotomus stantoni* is a widespread species in SE Asia, which seems not restricted to the karstic habitat, as it was recorded from different environments in neighboring countries of Laos [38, 42].

*Phlebotomus argentipes* was first described in India, and its geographical variety in terms of morphology and biology has been discussed [18, 46]. The recorded *Ph. argentipes* in Laos as well as in other SE Asian countries may be confused with closely related species, i.e., *Ph. mascomai*. A wide-scale study including many specimens studied by both morphology and genetics should be welcome. *Phlebotomus argentipes* was not found in the present study.

#### Genus *Sergentomyia*

The genus *Sergentomyia* is considered a catch-all group. As its role in the transmission of pathogens is quite limited, it has been less studied than the genus *Phlebotomus*. Some subgenera seem to be monophyletic, i.e., *Sergentomyia* sensu stricto, *Sintonius*, *Capensomyia*, *Vattieromyia*, *Trouilletomyia*, or *Ranavalonomyia.* The taxonomy of the genus *Sergentomyia* remains quite confusing, and the validity of some subgenera seems questionable to us, i.e., *Neophlebotomus* or *Parrotomyia*. Moreover, some species remain dubious, and the boundaries of other species are quite vague.

We report *Se. anodontis* sensu lato for the first time in Laos. *Sergentomyia anodontis* s.s. was described by Quate and Fairchild from Batu Cave in peninsular Malaysia. The main characteristics of this species were described as follows: “an unarmed cibarium, bur with spine-like projections from fold in membrane above sclerotized part and with median projection over which is inverted V-shaped bar, and spermatheca long and tubular, no differentiation between spermatheca and duct until point of junction of individual duct, apex with thick, hairy, sunken knob, several annulations below knob” [47]. The originality of its cibarium makes it very easily identifiable. For this reason, this species is not observed in detail in routine, and it seems to us that *Se. anodontis* s.l. could actually constitute a species complex. The specimens found in this study agree with this description. However, among the samples in this study, a pair of ascoids was found on flagellomere 1 (f1) to f13, which is different from the original description where a pair of ascoids found on f1 to f11 and a single ascoid on f12 to f14 have been observed. Different cibarial teeth were also observed in specimens from Vietnam [42]. Moreover, a recent genetic study of this species using cytochrome c oxidase I (*COI*) in Thailand showed that this species was divided into two clusters [48]. The genetic analysis using *Cyt-b* in this study showed that our samples were identical to some specimens from Thailand [27]. However, the number of sequence samples used for our analysis is small, and further larger-scale studies are needed to clarify the taxonomy status of this probable complex of species. For this study, we considered this species as *Se. anodontis* s.l.

*Sergentomyia bailyi* s.l. was recorded in Laos by Quate from burned tree holes [24] and was not found in the present study. As already discussed by Vu et al. [42] on sand flies from Vietnam, the reports of this species in Laos as well as other SE Asian countries still need to be revised by studying more samples and genetic studies.

The *Se. barraudi* group was also recorded in Laos by Quate [24]. The *Se. barraudi* group should be further studied because of the existence of considerable morphological and molecular heterogeneity in the morphological characters, such as the number and distribution of teeth on the cibarium. The cibarium in the original description made [49] from Indian specimens included 40 teeth and a forked anterior part of the sclerotized area. The specimens caught in Laos exhibited a similar forked sclerotized area and 50 to 54 cibarial teeth. Consequently, we identified the specimens of the present study as *Se. siamensis* considering that both morphological characters (Fig. 2A, D, G, J) and molecular markers were similar to those reported and already discussed in Thailand [27]. The results of the *Cyt-b* analysis in this study, together with the results of the COI analysis of the *Se. barraudi* group in Thailand [48], highlights the need for further taxonomic investigation in the SE Asian region. A complete review of the *barraudi* group is highly desirable. It will have to include numerous populations from various countries and combine morphological and molecular approaches.

*Sergentomyia rudnicki* is closely related to *Se. brevicaulis* and *Se. barraudi* group in presenting of comb-like teeth on cibarium. The *Se. barraudi* group could be separated from *Se. rudnicki* and *Se. brevicaulis* by their shorter antennae flagellomere 1, sclerotized area of the cibarium, and ovoid and smooth spermathecae (Fig. 2A and J). The specimens classified as *Se. rudnicki* from the present study agree with the original description of *Se. rudnicki* by female cibarium presenting about 90 comb-like teeth, two rows of about 20 vertical teeth, long pharyngeal teeth oriented backwards, and oblong and annulate spermatheca [18]. However, among the Laotian specimens, about 80 comb-like teeth on the cibarium with two rows of vertical teeth of more than 30 on each row were observed (Fig. 2F). Regarding *Se. brevicaulis*-like found in this study, cibarium has about 50 comb-like teeth (Fig. 2E), which is similar to the original description of *Se. brevicaulis* [24], but pharyngeal teeth long (Fig. 2H) like those in *Se. rudnicki* (Fig. 2I). Further studies are needed to clarify the taxonomic status of *Se. rudnicki* and the *Se. brevicaulis* group.

*Sergentomyia dvoraki* n. sp. is a species belonging to the *iyengari* group of *Sergentomyia*. Historically, *Se. iyengari* Sinton was described in 1933 from specimens caught in Southwest India without anterior teeth (formerly fore-teeth) and without any description of spermatheca [50]. Later, in 1935–1936, Raynal redescribed both the female and male of *Se. iyengari* Sinton using specimens from Vietnam. Raynal also illustrated both anterior teeth and wrinkled spermathecae [51] without suspecting that the samples from Vietnam could be different from those from Southwest India, making the situation confusing. For us, the description by Raynal of *Se. iyengari* from Vietnam may probably be the species that we described here. *Sergentomyia dvoraki* n. sp. could easily be separated from *Se. iyengari* because the latter species does not exhibit anterior teeth on the cibarium. The recently described *Se. ashwanii* [52] from India has a spermatheca very different from that of *Se. dvoraki* n. sp., without the terminal knob embedded in a deep notch. *Sergentomyia dvoraki* n. sp. has cibarial teeth similar to those in *Se. hivernus* by presenting 2–12 anterior teeth (Fig. 3B and C) but can be distinguished from each other by spermathecae that are tubular without limits between the body and duct in *Se. hivernus* [53], as shown in Fig. 3E and F. *Sergentomyia hivernus* was also historically wrongly considered a junior synonym of *Se. iyengari* Sinton in SE Asia. After more samples became available and additional genetics were analyzed recently, the previous records of *Se. iyengari* in Laos may refer to *Se. hivernus*. The spermathecae of *Se. dvoraki* n. sp. are similar to those of *Se. khawi* but with wrinkled bodies, and longer and narrower individual ducts than those of *Se. khawi* (Fig. 3D and F). In *Se. khawi,* the cibarium has many anterior (vertical) teeth arranged along 2–3 rows [54] (Fig. 3A). *Sergentomyia gemmea* can be easily separated from other species by the ascoids on antennae with spurs (Fig. 3I) but absent in *Se. khawi*. The records of *Se. gemmea* [18] in Laos are pending revision, as the cibarium appears to be different, and the pairwise distance of the *Cyt-b* gene between specimens from Laos and Thailand specimens was 0.06 [22]. A complete review of the *iyengari* group is highly desirable. It will have to include numerous populations from various countries and combine morphological and molecular approaches.

*Sergentomyia phasukae* recorded in Laos in this study has the same characteristics as those described by Curler [55]. This species is closely related to *Se. quatei*. Our specimens had no pigment patch on the cibarium (Fig. 3G). The arrangement of cibarial teeth looks somewhat different from the original description, but we think this is because of the mounting. The common spermathecal duct is as long (Fig. 3J) as in the original description of *Se. phasukae*.

*Sergentomyia sylvatica* was recorded in Laos by Quate in Vientiane collected from tree holes [24]. In this study, *Se. sylvatica* was found in all cave locations in karstic limestone in Vientiane Province. The spermathecae and cibarial (Fig. 3H and K) agree with the original description of this species in Vietnam by Raynal [56].

*Sergentomyia perturbans* was recorded in Laos by Quate as *Phlebotomus* (*Se.*) *sylvestris* collected from tree holes [24]. Later, this species was synonymized with *Se. perturbans*. As already discussed by Vu et al. [42] on sand flies from Vietnam, the taxonomic status of *Se. perturbans* remains doubtful. The record of this species in Laos should be considered as uncertain. We followed the morphological characteristics described by Lewis [18]: “cibarium with eight or nine distinct pointed teeth merging into about 10 spicules on each side, arising from thick refractive band, fore-teeth absent, pigment patch dark reddish brown anteriorly and grey posteriorly, with transverse and oblique line, bearing anteriorly about eight longitudinal lines; distinct cibarial bulge present. Pharynx less than twice as wide posteriorly as anteriorly, with faint ridges bearing minute spicules.” Images of the cibarium and pharynx of the Laotian samples are shown in Fig. 4A, D, and F.

*Sergentomyia marolii* n. sp. is morphologically closely related to *Se. bigtii* [57] from the Philippines, as indicated by the original description of the arrangement of 10 to 12 teeth on the cibarium and long teeth on the pharynx (Fig. 4B and E). However, this species can be easily distinguished from *Se. bigtii* by its shorter antennae f1. The metafurca of this species is strongly pigmented, similar to that of the specimens of *Se. perturbans* included in this study (Fig. 4C and F). It is interesting to further investigate whether this character could be used at subgenus level. Only two specimens were found during this study in Vientiane Province. Both were mounted in toto, and no DNA sequence is available for this new species.

#### Genus *Grassomyia*

*Grassomyia indica* s.l. was first reported in Laos from Vientiane and Luangphabang provinces as *Phlebotomus* (*Se.*) *squamipleuris* by Quate [24]. The genus *Grassomyia*, also considered as a subgenus of *Sergentomyia* by several authors without consensus, is not implicated in the transmission of pathogens, and consequently, its taxonomy remains poorly documented. In this study, we consider our specimens as *Gr. indica* s.l. because we assume that our specimens from Laos, and more broadly from SE Asia, probably belong to a species other than that described from India, but this hypothesis must be justified by a global morphological and molecular study including all *Grassomyia* species, as discussed previously [42]. In the present study, *Grassomyia* specimens were found mainly from cave entrances in Feung district. Their morphology and genetics are similar to those reported from Thailand [58] and may be as those that found in Vietnam, as the number of cibarial teeth ranged from 25 to 33.

## Conclusions

This study highlighted the high diversity of phlebotomine sand flies fauna in Laos, which was previously underestimated in karstic limestone areas. However, the taxonomic status of many species in Laos, as well as in other countries of Southeast Asia, still needs more in-depth study using both morphological characters and molecular methods. Many species could be found from inside, at the entrance, and outside of caves, indicating a wide range of host-seeking behavior in the karstic cave areas.

## Supplementary Information

Additional file 1: Table S1: Characteristics of sand fly collection locations in karstic areas of Vientiane Province.

Additional file 2: Table S2: Details and source of sequences used in this study.

Additional file 3: Table S3: The pairwise distance of cytochrome b gene within species and between species found in this study.

Additional file 4: Fig. S1: Shannon diversity indices (*H*) and species accumulation curves for the sand flies examined in this study. Shannon indices by district (A), by trapping position (B), and by season of collection (C). Species accumulation curves by district (D), by trapping position (E) and by season of collections (F).

Additional file 5: Fig. S2: The relative abundance of sand flies by district (A) and sampling location (B). Principal coordinate analysis based on the Bray–Curtis index showing the species assemblage between districts (C) and sampling locations (D). *PNK* Tham Phanokkok cave, *TN* Tham Nam cave, *TP* Tham Pha cave, *TY* Tham Yao cave, *PLS* Tham Phaluesy cave, *NOK* Tham Nang Oau Khiem cave, and *AL* Angluang karstic areas.

Additional file 6: Table S4: Sand fly species composition from different sampling locations from three districts of Vientiane Province in 2019. *PNK* Tham Phanokkok cave, *TN* Tham Nam cave, *TP* Tham Pha cave, *TY* Tham Yao cave, *PLS* Tham Phaluesy cave, *NOK* Tham Nang Oau Khiem cave, and *AL* Angluang karstic areas.

Additional file 7: Table S5: Species composition of sand flies by trapping positions.

Additional file 8: Table S6: Species composition of sand flies by trapping season among collection districts.

## Data Availability

All specimens collected during this study were examined and deposited at the Institut Pasteur du Laos Collection Room. Sequences of *Cyt-b* obtained from this study were deposited in the GenBank database (PQ151806–PQ151934). No datasets were generated or analyzed during the current study.

## Abbreviations

AL: Angluang karstic areas
COI: Cytochrome c oxidase I
*Cyt-b*: Cytochrome b
f1–f14: Flagellomere 1–14
NOK: Tham Nang Oau Khiem cave
PLS: Tham Phaluesy cave
PNK: Tham Phanokkok cave
r1–r5: Radial wing vein branches 1–5
ti–v: Tarsomere i–v
TN: Tham Nam cave
TP: Tham Pha cave
TY: Tham Yao cave
