# Supplementary figures and images for: A study on the diversity of phlebotomine sand flies (Diptera, Psychodidae) in karstic limestone areas in Vientiane Province, Laos, with a description of two new species of Sergentomyia França and & Parrot
Source: Parasit Vectors. 2024 Sep 11;17:385. doi: 10.1186/s13071-024-06444-w (PMC11389125; doi:10.1186/s13071-024-06444-w)

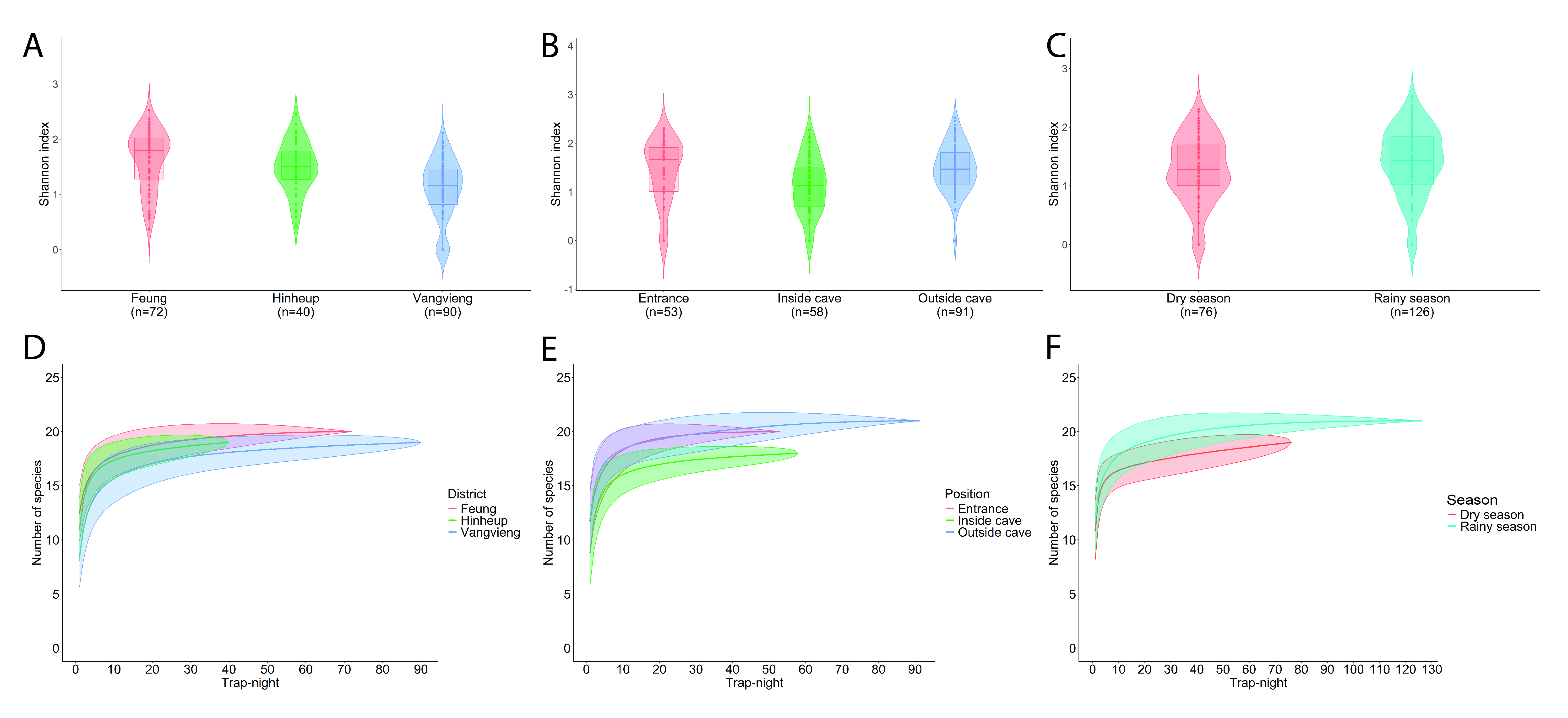

Supplement: Supplementary file 4 — Additional file 4: Fig. S1: Shannon diversity indices (H) and species accumulation curves for the sand flies examined in this study. Shannon indices by district (A), by trapping position (B), and by season of collection (C). Species accumulation curves by district (D), by trapping position (E) and by season of collections (F). [file 13071_2024_6444_MOESM4_ESM.jpg]

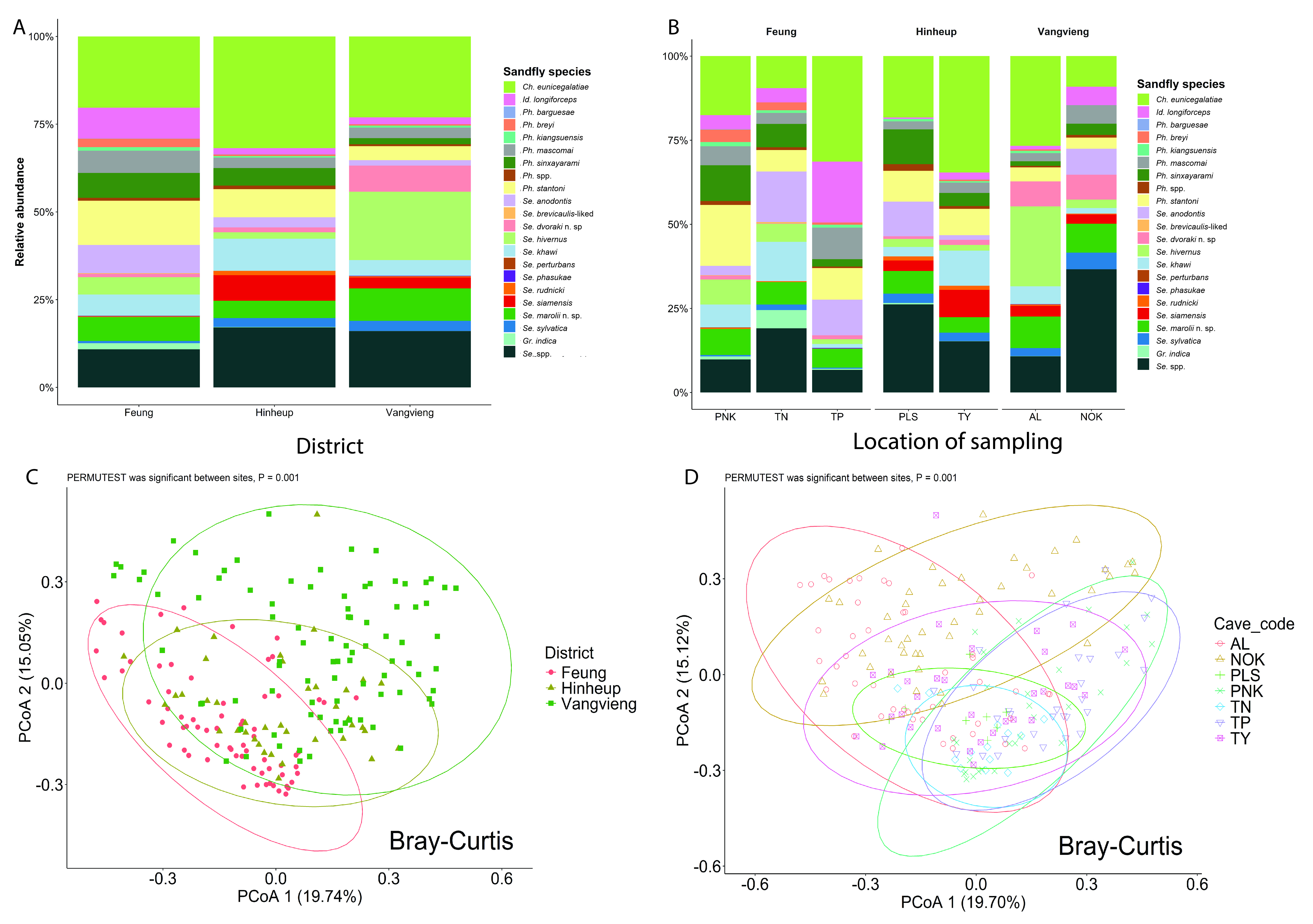

Supplement: Supplementary file 5 — Additional file 5: Fig. S2: The relative abundance of sand flies by district (A) and sampling location (B). Principal coordinate analysis based on the Bray–Curtis index showing the species assemblage between districts (C) and sampling locations (D). PNK Tham Phanokkok cave, TN Tham Nam cave, TP Tham Pha cave, TY Tham Yao cave, PLS Tham Phaluesy cave, NOK Tham Nang Oau Khiem cave, and AL Angluang karstic areas. [file 13071_2024_6444_MOESM5_ESM.jpg]
